# Prediction of Flow Effect on Crystal Growth of Semi-Crystalline Polymers Using a Multi-Scale Phase-Field Approach

**DOI:** 10.3390/polym9120634

**Published:** 2017-11-23

**Authors:** Xiaodong Wang, Jie Ouyang, Ying Liu

**Affiliations:** 1 School of Mathematical Sciences, Peking University, Beijing 100871, China; 2 Department of Applied Mathematics, Northwestern Polytechnical University, Xi’an 710129, China; 3 School of Arts and Sciences, Shaanxi University of Science & Technology, Xi’an 710021, China

**Keywords:** crystal growth, crystallization, orientation, semi-crystalline polymer, flow effect, phase-field, multi-scale

## Abstract

A multi-scale phase-field approach, which couples the mesoscopic crystallization with the microscopic orientation of chain segments and macroscopic viscoelastic melt flow, is proposed to study how the crystal growth of semi-crystalline polymers is affected by flows. To make the simulation feasible, we divide the problem into three parts. In the first part, a finitely extensible nonlinear elastic (FENE) dumbbell model is used to simulate the flow induced molecular structure. In the second part, formulas for estimating the density, orientation and aspect ratio of nuclei upon the oriented molecular structure are derived. Finally, in the third part, a massive mathematical model that couples the phase-field, temperature field, flow field and orientation field is established to model the crystal growth with melt flow. Two-dimensional simulations are carried out for predicting the flow effect on the crystal growth of isotactic polystyrene under a plane Poiseuille flow. In solving the model, a semi-analytical method is adopted to avoid the numerical difficult of a “high Weissenberg number problem” in the first part, and an efficient fractional step method is used to reduce the computing complexity in the third part. The simulation results demonstrate that flow strongly affects the morphology of single crystal but does not bring a significant influence on the holistic morphology of bulk crystallization.

## 1. Introduction

As is well known, crystallization of polymers is very different from that of the small molecular materials. Generally, the crystallization of polymers is more complex, and no polymer can be fully crystallized. The final morphology and crystallinity of polymers strongly depend on both their interior structures (molecular configuration and chain conformation) and exterior conditions. Because the resulting crystalline structures have significant impacts on material properties and further determine the performance of products in end uses, the research of polymer crystallization has great significance.

After extensive study over a long period in the past, the research findings and theories about polymer crystallization in quiescent conditions are on the way to perfection. However, under real processing conditions (such as injection molding, extrusion molding, fiber spinning, and etc.), polymers always undergo the force (shearing, stretching or extrusion) of external fields. In this case, the crystallization behavior of polymers will be greatly changed. It has been known that flow shortens the nucleation time, enhances the nucleation density and accelerates the crystal growth of polymers [1,2]. Moreover, flow also changes the crystallization morphology and produces more plentiful crystal patterns than quiescent conditions, such as columnar crystals, shish-kebab crystals, fibrillar crystals, etc. [3,4,5,6]. In view of the importance of morphology to performance, the effects of flow on morphology during the crystal growth period will be investigated in this paper.

These years, numerical simulation has been necessary, complementary to the experimental study of polymer crystallization. Plenty of simulations using approaches ranging from simple expressions to multi-scale models have been performed to predict the flow effect on crystal growth of semi-crystalline polymers. Rong et al. [7] presented a multi-scale model for simulating the isothermal flow-induced crystallization of polymer melt in a simple shear flow. A finitely extensible nonlinear elastic (FENE) dumbbell model and a rigid dumbbell model are used to describe the amorphous phase and semi-crystalline phase, respectively. The model takes into account the rheological behavior of polymer melt and limits to the spherulitic growth. Mu et al. [8] studied the crystallization and orientation of polymer melts with amorphous and semi-crystalline phases undergoing processing conditions by using a two-phase model. The rheological behavior of polymer melt in this model is involved by an approach similarly with the one used in [7]. Based on the proposed mathematical model and numerical algorithm, the crystallization and orientation of polymer melts in the hollow profile extrusion process were predicted. Spina et al. [9] implemented a robust framework for the computation of the crystallization kinetics of thermoplastic polymer by using a multi-scale approach. The multi-scale modeling assesses parameters influencing microstructure formation though experiments rather than time-consuming analysis. The research shows that the framework is able to reproduce the crystallization kinetics under non-isothermal and temperature-gradient conditions. Although plenty of studies have been carried out, the consideration about flow effect on the crystal morphology is still insufficient. Because the present multi-scale models are mainly built based on the fundamental theories of crystallization kinetics and rheology of semi-crystalline polymers, they are incapable of predicting flow effect on crystallization morphology together with microstructure of the crystals. Accurate prediction of flow effect on crystal growth of semi-crystalline polymers still requires not only appropriate mathematical models that consider the characteristics of polymer melt but also robust numerical methods for solving the models.

In morphological research, there have been many simulation approaches available, which include molecular dynamics methods [10,11,12,13], coarse-grained Monte Carlo methods [14,15,16,17,18,19], mesoscopic methods [20,21,22,23,24,25] and phenomenological methods [26,27,28,29]. Among these methods, the molecular dynamics methods are at the highest level of detail. They can provide a rigorous approach to crystallization by resolving the motion of individual monomers with very few modeling assumptions. However, these methods suffer from strong lengthscale and timescale limitations. The coarse-grained Monte Carlo methods significantly reduce the computational cost and hugely increase the simulation time step. This allows larger chains to be simulated for longer timescales compared to the first class of methods. Although these methods have made significant progress, they are still limited by their computational cost and require coarse-graining assumptions. The mesoscopic methods can be regarded as a trade-off between microscopic methods and macroscopic methods. Despite many assumptions, these kinds of methods can obtain simulation results agreeing well with the experimental observations and thus give qualitative descriptions for the crystal growth of polymer melt. They have the ability to reveal the formation mechanisms of crystallization morphology by moderate computational cost. One of the most prevalent approaches in this kind is the phase-field method [22,23,24,25]. Finally, phenomenological methods have their advantage in getting rid of the restriction of computational expenses on the accessible lengthscales and timescales. These methods can also give qualitative predictions, but their fatal shortcoming is the lack of physical basis compared to the mesoscopic methods. In view of the excellent performance of the phase-field method, it is adopted in this work to simulate the crystal growth of semi-crystalline polymers under flow.

The phase-field method uses a crystal order parameter to describe phase transition. Evolution of the crystal order parameter can be governed by a group of nonlinear partial differential equations derived from the Ginzburg–Landau theory. So far, this method has gained great success in understanding the dendritic growth of small molecular materials [30,31,32,33,34], spherulitic growth of polymers [22,23,24,25,35,36,37] and shish-kebab growth of polymers [38]. However, as to the coexistent system of viscoelastic flow and polymer crystallization, the phase-field method is still in the blank stage. Thus, the present paper aims at generalizing the phase-field method to the flow induced crystal growth of polymers and revealing the corresponding mechanism.

In fact, much effort has been made on simulating the coexistent system of flow and dendritic growth of metals by the phase-field method [39,40]. However, polymer crystallization is rather different and more complex due to the long chain structure. Directly extending the existing studies for metal to polymer is infeasible. The specific reasons for this may be summarized as follows. Firstly, there is no need to consider the shape of each nucleus in these studies, and point-like nuclei are generally adopted in simulations. In contrast, under flow conditions, polymer chain segments in melt would be extended from random coil conformation into microfibrillar structure along flow direction [41]. The oriented molecular structure can survive a long time after the weakening or cessation of flow [42,43,44], and helps to form oriented nuclei during the subsequent solidification. Because the oriented nuclei can remarkably change the crystal growth of semi-crystalline polymers [45], the shapes of nuclei must be considered in modeling the flow induced crystal growth of polymers. Secondly, the existing studies for metal do not need to consider elasticity of the melt. However, polymer melt is a typical viscoelastic fluid and elasticity of the melt may have a potential effect on the crystal growth [46]. Thirdly, these studies do not consider the change of fluid property during solidification. However, research on crystallization rheology elucidates that crystallization has a remarkable influence on the rheological property of polymer melt [47]. Thus, the change of fluid property cannot be ignored in modeling the flow induced crystal growth of polymers. In this paper, for solving these problems, a multi-scale phase-field model, which links the mesoscopic crystal growth with the macroscopic flow and microscopic molecular structure, is established to predict the flow effect on crystal growth of semi-crystalline polymers.

The rest of this paper is arranged as follows. In Section 2, theoretical description of our multi-scale phase-field approach for modeling the flow induced crystal growth is given. The numerical method for solving the proposed model is given in Appendix A, Appendix B, Appendix C and Appendix D. In Section 3, the proposed model and numerical method are used to predict flow effect on crystal growth of isotactic polystyrene in a plane Poiseuille flow. Finally, some concluding remarks are given in Section 4.

## 2. Mathematical Formulation

The physical problem considered is first putting the hot polymer melt into a fast simple flow for a period of time, and then decreasing the flow to a large extent and cooling the melt to a scheduled crystallization temperature quite rapidly. This process may be divided into three stages, which are molecular stretch and orientation by fast flow, nucleation upon the flow induced structure and crystal growth with slow melt flow. For this reason, our mathematical model includes three parts accordingly. Details for this are showing below.

### 2.1. Modeling Flow Induced Molecular Structure

When polymer solution or melt is undergoing the shear or elongation of fast flow, the whole chain or chain segments of a molecule will have the opportunity to be stretched and oriented. The stretched and oriented structures would be completely or partly retained after the weakening or cessation of flow, and then have important effect on the subsequently crystallization. Several computational models have been available for predicting the molecular orientation of polymer undergoing flow, such as the Leonov model [48], the FENE dumbbell model [49], etc. In view of the widely and maturely application of the FENE dumbbell model with a Peterlin closure approximation (FENE-P model), it is adopted in this work.

The FENE-P model is developed from the dumbbell model of polymer molecules. A dumbbell, which consists of two rigid beads connected by a massless spring, is an abstract representation of a polymer molecule. At any given time, the beads are subjected to friction force, Brownian force and spring force. According to Newton’s second law and neglecting the acceleration, the equation for the relative motion of the beads would be obtained as

(1) $$\frac{dQ}{dt}={\left(\nabla u\right)}^{T}\cdot Q-\frac{2k_{B}T}{\zeta }\frac{\partial }{\partial Q}\ln\phi -\frac{2}{\zeta }F$$

where Q is the end–to–end vector of the dumbbell, u is the velocity, $k_{B}$ the Boltzmann constant, T the thermodynamic temperature, ζ the friction coefficient, ϕ the distribution function and F the spring force. On the other hand, ϕ should satisfy the condition of continuity in the conformation space. That is,

(2) $$\frac{\partial \phi }{\partial t}+\frac{\partial }{\partial Q}\cdot \left(\frac{dQ}{dt}\phi \right)=0$$

The substitution of dQ/dt from the equation of motion (1) into the equation of continuity (2) gives the following diffusion equation:

(3) $$\frac{\partial \phi }{\partial t}+\frac{\partial }{\partial Q}\cdot \left({\left(\nabla u\right)}^{T}\cdot Q\phi \right)-\frac{2k_{B}T}{\zeta }\frac{\partial^{2}\phi }{\partial Q^{2}}-\frac{2}{\zeta }\frac{\partial }{\partial Q}\cdot \left(\phi F\right)=0$$

Once the flow velocity u and spring force F are given, then the diffusion equation can, in principle, be solved to get the conformation distribution of dumbbells. For the FENE dumbbell model, the spring force in Equation (3) is given by

(4) $$F=\frac{HQ}{1-(Q^{2}/Q_{0}^{2})}$$

where H is the elastic coefficient, Q=|Q| is the length of spring, and $Q_{0}$ is the maximum extensibility.

Generally, directly solving Equation (3) is rather difficult, so the moment-closure approach is very popular and adopted in this work. The equation for the second moments reads

(5) $$\frac{D\langle QQ\rangle }{Dt}-{\left(\nabla u\right)}^{T}\cdot \langle QQ\rangle -\langle QQ\rangle \cdot (\nabla u)=\frac{4k_{B}T}{\zeta }I-\frac{4}{\zeta }\langle QF\rangle$$

where D/Dt=∂/∂t+u⋅∇ is the material derivative, I is the unit tensor, and the second moments 〈QQ〉 represents the conformation tensor. For the FENE-P model, the spring force in Equation (5) is approximated as

(6) $$F=\frac{HQ}{1-\langle Q^{2}/Q_{0}^{2}\rangle }$$

By inserting Equation (6) into Equation (5) and defining the dimensionless conformation tensor $C=H\langle QQ\rangle /k_{B}T$, we get the following evolution equation:

(7) $$\lambda \left(\frac{DC}{Dt}-{\left(\nabla u\right)}^{T}\cdot C-C\cdot (\nabla u)\right)=-\frac{C}{1-tr(C)/b}+I$$

where $b=HQ_{0}^{2}/k_{B}T$ is the dimensionless maximum extensibility and λ=ζ/4H is the molecular relaxation time.

By employing the dimensionless conformation tensor C, the information of molecular orientation and stretch would be obtained [50]. From the definition, it is obvious that C is a symmetric positive definite second-rank tensor. This ensures that we can always get the positive eigenvalues and real eigenvectors of the conformation tensor. If we regard the directions of the eigenvectors as the major and minor axes and the eigenvalues as the corresponding lengths, then an orientation ellipse for 2D or an orientation ellipsoid for 3D will be obtained. Specifically, when the polymer is in its equilibrium state (without flow), we have $C=b/(b+d_{s})I$, where $d_{s}=2,3$ is the dimension of space. In this situation, the orientation ellipse or ellipsoid degenerates to orientation circle or sphere, which means that the orientation probability along any direction is the same. Otherwise, the orientation along the major axis will be dominant. In this work, we simply deem that the molecule is always oriented along the major axis of the orientation ellipse or ellipsoid when it is undergoing flow.

In addition, the conformation tensor may also be used to quantify the molecular stretch. Generally, the first invariant tr(C) is treated as a measure of molecular stretch. In an equilibrium state, we have tr(C)=2b/(b+2) for 2D. In a non-equilibrium state, the deformation of the molecular chain will be increasing as the shear or elongation is intensified, and accordingly tr(C) will trend to the maximum extensibility b. The amount of the molecular stretch tr(C) plays an important role in polymer crystallization. It is an intermediate cause of the anisotropic nucleation, and hence the cause of the anisotropic crystallization morphology.

Based on the above, once C is solved from Equation (7), then the flow induced molecular structure would be quantitatively described. For some simple flow fields, the velocity u can be analytically solved in the equilibrium states. In these cases, Equation (7) is immediately closured when substituting the steady expression of u. However, for general flow fields, the velocity u must be solved from the Navier–Stokes equations. In these cases, the transient interaction between macroscopic flow field and microscopic molecular motions cannot be ignored. From Equation (7), we know that the effect of flow on molecular motions has been considered. In turn, the molecular motions affect flow mainly through changing the rheological behavior of the polymer fluids, and this can be embodied by the extra stress tensor. In the “Giesekus form”, the extra stress tensor of polymer fluid is given as

(8) $$\tau_{p}=\frac{\eta_{p}}{\lambda }\left(\frac{C}{1-tr(C)/b}-I\right)$$

where $\eta_{p}$ is the viscosity of polymer fluid. By this time, the velocity u can be solved through coupling Equations (7) and (8) with the incompressible Navier–Stokes equations:

(9) ∇⋅u=0

(10) $$\rho (\frac{\partial u}{\partial t}+u\cdot \nabla u)=-\nabla p+\eta_{s}\Delta u+\nabla \cdot \tau_{p}$$

where ρ, p and $\eta_{s}$ are the density, pressure and viscosity of solvent, respectively. In physics, Equation (9) represents the conservation of mass and Equation (10) represents the conservation of momentum. Because the stage considered in this subsection is under isothermal conditions, the energy equation is not included in the Navier–Stokes equations.

### 2.2. Modeling Nucleation upon Oriented Molecular Structure

It has been well known that flow has great influence on the crystallization of polymer, especially on the nucleation stage. Stable nuclei or precursors with some degree of order can often be generated from polymer melt that has just experienced flow history. Generally, flow affects the nucleation stage of polymer crystallization by providing both extra nucleation sites and oriented templates. The former accelerates kinetics of the crystallization and the latter induces anisotropic crystal growth. The following content in this subsection will focus on seeking mathematical descriptions for the two aspects.

Much evidence has shown that the number of activated nuclei formed during solidification from the melt with flow history is usually greater than that without flow history. Based on the experimental observations, the total nuclei may be divided into two parts, which are the general nuclei observed in quiescent condition $N_{q}$ and the additional nuclei appearing after flow $N_{f}$. Thus, the density of activated nuclei N may be written as the sum of $N_{q}$ and $N_{f}$, viz.,

(11) $$N=N_{q}+N_{f}$$

In quiescent conditions, the following equation, which describes the nucleation density as a function of temperature, is generally used in literature to evaluate $N_{q}$. That is,

(12) $$N_{q}(T)=N_{0}\exp\left(\varphi \cdot (T_{m}-T)\right)$$

where $T_{m}$ is the melting temperature, and $N_{0}$ and φ are empirical parameters. On the other hand, according to the work of Koscher and Fulchiron [51], the number of additional nuclei appearing after flow treatment was linked to the first normal stress difference $N_{1}$. The simplest mathematical relationship between $N_{f}$ and $N_{1}$ is

(13) $${\dot{N}}_{f}=CN_{1}$$

where C is a scale factor determined from experiments.

For two-dimensional problems, the 3D nucleation density giving in Equation (11) should be converted to a 2D nucleation density. Referring to the work done by Charbon and Swaminarayan [52], the stereological relationship

(14) $$N_{2D}=1.458{\left(N_{3D}\right)}^{2/3}$$

is adopted in this study. Because the influence of flow on the crystal growth rate is less relevant than on the nucleation process [47], the explicit consideration of flow effect on the kinetics of polymer crystallization is limited to the above.

As to the oriented templates, the flow induced molecular structure plays a very important role. It is generally accepted that the ordered (stretched and oriented) structures generated by flow is responsible for the oriented nucleus formation and hence the subsequently anisotropic crystal growth. Different crystalline patterns or morphologies may be produced depending on the molecular orientation and stretch prior to the start of crystallization. Because our attention in this work is mainly paid to the crystalline morphology, it is necessary to establish the relationships between the ordered structures and oriented nuclei.

In theoretical modeling, the oriented nucleus is usually regarded as possessing a cylindrical appearance for simplicity’s sake [41]. This way, any oriented nucleus can be described by two characters, which are orientation and aspect ratio. In the next content, our task will be quantitatively giving descriptions of these characters. To keep it simple in this paper, we assume that the ordered structures induced by flow would be completely retained after the weakening or cessation of flow until the nucleation takes place. Based on this assumption, the orientation of one nucleus may be directly set as the molecular orientation at the place where it is located. Accordingly, the aspect ratio of the nucleus, which is associated with the amount of molecular stretch tr(C), is determined by the following equation:

(15) $$l_{w}=1+M\sqrt{1-{d_{s}}^{d_{s}}det\left(\frac{C}{tr(C)}\right)}tr(C)$$

where the coefficient M depends on the crystallization property of the polymer. For 2D case, the eigenvalues and eigenvectors of C can be calculated by

(16) $$\lambda_{1,2}=\frac{tr(C)\pm \sqrt{{\left(tr(C)\right)}^{2}-4det(C)}}{2}$$

(17) $$\xi_{1,2}={\left(1,\;\frac{\lambda_{1,2}-c_{11}}{c_{12}}\right)}^{T}$$

for $c_{12}\neq 0$ and

(18) $$\lambda_{1}=c_{11},\;\lambda_{2}=c_{22}$$

(19) $$\xi_{1}={\left(1,\;0\right)}^{T},\;\xi_{2}={\left(0,\;1\right)}^{T}$$

for $c_{12}=0$. Thus, Equation (15) in 2D can be rewritten as

(20) $$l_{w}=1+M\sqrt{1-\frac{4det\left(C\right)}{{\left(tr(C)\right)}^{2}}}tr(C)=1+M\left|\lambda_{1}-\lambda_{2}\right|$$

Compared to Equations (15) and (20) provides a more intuitive expression. It actually uses the “aspect ratio” of the orientation ellipse to describe the aspect ratio of the nucleus. Moreover, this equation also means that $\Delta =\lambda_{\max}-\lambda_{\min}$ can be treated as another measure of molecular stretch. Recently, this measure is adopted by Pantani et al. in modeling morphology evolution during polymer crystallization under processing conditions [53].

In this work, we will model the crystal growth of semi-crystalline polymer upon oriented nuclei by using phase-field approach. At present, the studies of crystal growth based on phase-field methods mainly consider the point-like nuclei. However, it is lucky that we have successfully developed the oriented nuclei into phase-field modeling in our recent work [38]. For this study, the nuclei used in simulation will be more complicated. Their shapes are dependent on the flow history prior to nucleation events. Point-like and oriented nuclei may appear together in one simulation. For generating a nucleus, we should first evaluate its orientation and aspect ratio according to the flow history at the position where the nucleus is located, and then “create” it on the lattice by using the method given in our previous work [38].

### 2.3. Modeling Crystal Growth with Melt Flow

The formation of morphological features in solidification of semi-crystalline polymers under flow conditions has been investigated over many years. Through experimental observations, many flow induced crystallization patterns that are very different from those obtained from quiescent conditions have been found. However, little has been known about the influence of flow on microstructure development. Generally, when crystal growth is accompanied by slow melt flow, not only does flow influence the crystallization pattern, but the evolving microstructure can also trigger unexpected and complicated flow phenomena. Therefore, it is necessary to develop a flow and crystal growth coupled model to study the complex interaction between melt flow and morphology evolution of polymers.

For modeling the coexistent system of crystal growth and melt flow of polymers, the existing relevant studies on small molecule materials may be used as a reference. At present, there are mainly two kinds of methodologies mostly used, which are developed by Anderson et al. [54,55,56] and Beckermann et al. [39,57], respectively. The former kind treats not only the liquid phase but also the solid phase as Newtonian fluids, and specifies the viscosity of the solid phase to be much larger than that of the liquid phase. While the latter kind assumes the solid phase to be rigid and stationary, and does not rely on specifying a variable viscosity across the diffuse interface region that tends to a large value in the rigid solid. Because the latter kind can be used with any diffuse interface technique without much particular modification, it is partly referenced in this work.

Let ψ denote the phase-field variable, where ψ=0 and ψ=1 refer to the bulk liquid phase and solid phase at equilibrium state, respectively. During solidification, the phase-field variable varies smoothly from zero in the liquid to unity in the solid in a narrow but numerically resolvable diffuse interface region. Since polymers are hardly to be fully crystallized, the value of ψ is impossible to reach unity for ordinary studied cases. According to the Ginzburg–Landau theory, the temporal evolution of ψ can be governed by

(21) $$\frac{\partial \psi (x,t)}{\partial t}=-\Gamma \frac{\delta F(\psi ,T)}{\delta \psi (x,t)}$$

where Γ is the interface mobility of the system, T is the temperature and F(ψ,T) is the total free energy of the system. In the phase-field modeling for the solidification, F(ψ,T) often consists of two parts, which are a local free energy density $f_{local}(\psi ,T)$ and a gradient free energy density $f_{grad}(\psi )$, viz.,

(22) $$F(\psi ,T)=\int \left[f_{local}(\psi ,T)+f_{grad}(\psi )\right]d\Omega$$

where Ω is the region occupied by the system. The local free energy density is given in the form of an asymmetric double well as follows [22]:

(23) $$f_{local}(\psi ,T)=W\int_{0}^{\psi }\phi (\phi -\zeta_{0})[\phi -\zeta (T)]d\phi$$

where W describes the height of energy barrier for surface nucleation, ζ(T) is the unstable energy barrier and $\zeta_{0}$ is the value of ψ relevant to the stable solidification potential. Following the work done by Kyu et al. [22], $\zeta_{0}$ may be simply estimated as $\zeta_{0}=T_{m}/T_{m}^{0}$, where $T_{m}$ and $T_{m}^{0}$ are, respectively, the experimental melting temperature and equilibrium melting temperature of polymers. The gradient free energy density describing the symmetric or asymmetric growth process can be written as

(24) $$f_{grad}(\psi )=\frac{1}{2}\kappa_{0}^{2}\beta^{2}(\theta ){\left(\nabla \psi \right)}^{2}$$

where $\kappa_{0}$ is the coefficient of interface gradient, θ is the orientation angle and β(θ) describes the anisotropic growth rate of the interface. In literature, β(θ) is often given as

(25) β(θ)=1+εcos(jθ)

with ε the strength of anisotropy and j the number of modes.

By substituting Equations (22)–(25) into Equation (21), one obtains the following phase-field equation for planar crystallizations:

(26) $$\begin{array}{cc} \frac{\partial \psi (x,t)}{\partial t}=-\Gamma & (W\psi (\psi -\zeta (T))(\psi -\zeta_{0})-\kappa_{0}^{2}\nabla \cdot \left(\beta^{2}(\theta )\nabla \psi \right)\\ & +\kappa_{0}^{2}\frac{\partial }{\partial x}\left(\beta (\theta )\beta^{\prime }(\theta )\frac{\partial \psi }{\partial y}\right)-\kappa_{0}^{2}\frac{\partial }{\partial y}\left(\beta (\theta )\beta^{\prime }(\theta )\frac{\partial \psi }{\partial x}\right)) \end{array}$$

Because the melt flow during solidification is usually very slow, migration of crystallized structure by flow may be neglected. Accordingly, we assume that the phase-field variable is not advected by flow, thus the phase-field Equation (26) is unchanged from the stationary diffusive case.

To determine the temperature at the growing crystal fronts, a heat conduction equation may be deduced from the conservation law of enthalpy. For simplicity, the liquid and solid are assumed to have the same density ρ, thermal conductivity $k_{T}$, specific heat capacity $C_{p}$, and latent heat of fusion ΔH. Under flow conditions, the phase-transition temperature is advected by flow and dependents on stress. Since the solid phase is assumed to be rigid and stationary, flow in the diffuse interface region will weaken with the phase-field variable. According to the work of Beckermann et al. [39,57], the “residual” flow in the diffuse interface region for polymers may be expressed as $[1-\psi /\zeta_{0}]u$ phenomenologically. It is obvious that this expression does not only hold in the diffuse interface region but also in other region. Therefore, the energy equation, including phase-field variable dependent advective flux term, can be written as

(27) $$\rho C_{p}\left(\frac{\partial T}{\partial t}+[1-\psi /\zeta_{0}]u\cdot \nabla T\right)=k_{T}\nabla^{2}T+\rho \Delta H\frac{\partial \psi }{\partial t}+\tau :\nabla ([1-\psi /\zeta_{0}]u)$$

where $\tau =\eta_{s}(\nabla u+\nabla u^{T})/2+\tau_{p}$ is the total flow induced stress.

For crystal growth and melt flow coexistent system, conservation equations for mass and momentum need to be valid not only in the solid and liquid phases, but also in the diffuse interface region where the solid and liquid phases coexist. During solidification, the phase-field variable ψ for polymers varies smoothly from zero in the bulk liquid to $\zeta_{0}$ in the solid, so $\psi /\zeta_{0}$ can be viewed as a volume fraction of solid and used to derive the averaged solid and liquid conservation equations. Under the incompressible assumption, the averaged continuity equation gives

(28) $$\nabla \cdot ([1-\psi /\zeta_{0}]u)=0$$

where $\left[1-\psi /\zeta_{0}\right]$ represents the volume fraction of liquid and u is the intrinsic liquid velocity. Similarly, the averaged momentum equation for polymers that are viscoelastic fluids takes the following form:

(29) $$\begin{array}{l} \rho \left(\frac{\partial ([1-\psi /\zeta_{0}]u)}{\partial t}+[1-\psi /\zeta_{0}]u\cdot \nabla u\right)\\ \;=-[1-\psi /\zeta_{0}]\nabla p+\eta_{s}\Delta ([1-\psi /\zeta_{0}]u)+\nabla \cdot ([1-\psi /\zeta_{0}]\tau_{p}) \end{array}$$

Note that Equation (29) is indeed absent in the solid phase. Recall that the solid is assumed to be stationary and rigid, such that a momentum equation for the solid phase is not needed. In order to solve Equation (29), a constitutive equation for calculating the extra stress tensor $\tau_{p}$ still must be involved. For doing this, we continue using the FENE-P model introduced in Section 2.1, which is often regarded as a molecular typing constitutive equation for describing the viscoelastic behavior of polymer fluids. By employing the above idea again, the averaged form of Equation (7) gives

(30) $$\lambda \left(\frac{DC}{Dt}-{\left(\nabla [1-\psi /\zeta_{0}]u\right)}^{T}\cdot C-C\cdot (\nabla [1-\psi /\zeta_{0}]u)\right)=-\frac{C}{1-tr(C)/b}+I$$

where the material derivative is modified to $D/Dt=\partial /\partial t+[1-\psi /\zeta_{0}]u\cdot \nabla$ accordingly. The extra stress tensor $\tau_{p}$ can now be calculated by using Equation (8) once C is solved out from Equation (30).

If the problem considered is a sharp interface one instead of the diffuse interface one, there would be no volume flow at the interface. That is to say, the flow has a no-slip condition between the melt and the solid. The work done by Beckermann et al. realizes this condition via a drag resistivity in the diffuse interface region [57]. In this study, another way, which relies on specifying a variable viscosity across the diffuse interface region, is adopted. Since the momentum equation has been averaged, the variation of viscosity would not bring much numerical difficulty, even though the viscosity would tend to a large value in the rigid solid.

So far, plenty of researches have demonstrated that the crystallization has a dramatic influence on polymer viscosity and this phenomenon must obviously be taken into account [58]. Despite of the importance of the subject, the relevant literature on the effect of crystallinity on viscosity is rather scarce. This might be due to the difficulties in measuring simultaneously rheological properties and crystallinity evolution during the same tests. Even so, most of the researchers essentially agree that melt viscosity experiences an abrupt increase with crystallinity degree. Based on the work of Katayama and Yoon [59], the following phase-field variable dependent viscosities may be obtained

(31) $$\eta_{s}=(1+a_{0}\psi )\eta_{s0}\;\mathrm{and}\;\eta_{p}=(1+a_{0}\psi )\eta_{p0}$$

where $a_{0}$ is an empirical parameter setting as 100 in this study. $\eta_{s0}$ and $\eta_{p0}$ are the original viscosities. At last, we assume that the viscosity is not dependent on temperature, for the reason that the temperature will not be changed much.

## 3. Results and Discussion

As we have stated at the beginning of Section 2, the physical problem we considered may be divided into three stages. Therefore, simulations are accordingly including three steps. The first step is to obtain the fast flow induced structure by solving the partial differential equation (PDE) system (7)–(10), the second step calculates the density, orientation and aspect ratio of the nuclei by the algebraic Equations (11)–(20) and generates the nuclei upon the simulation lattice, and the third step simulates the crystal growth with slow melt flow by solving the PDE system (26)–(31). Among the three steps, numerical difficulties often arise in the first and last steps. In Appendix A, Appendix B, Appendix C and Appendix D, we present our methods for these two steps and summarize the whole simulation process in detail.

Simulations are carried out in 2D for the models presented above using rectangular computation domains. The inlet velocity boundary conditions are imposed on the left side boundary and their values are $u=u_{0}$ and v=0. The model parameters used in simulations are $T_{c}=200$ °C, $T_{m}^{0}=242$ °C, $\zeta_{0}=0.953$, ζ=0.167, W=15.43, $\kappa_{0}^{2}=0.916$, α=0.658 and K=1.578. These parameters are calculated from a set of experimentally accessible physical parameters of isotactic polystyrene (iPS) at a crystallization temperature of $T_{c}=200{}^{\circ}C$ [50]. In addition, the dimensionless maximum extensibility of the molecular and empirical parameters for nucleation are assigned values b=200, $N_{0}=1.74\times 10^{12}/m^{3}$ and φ=0.155 [58]. We have used Δt=0.1, Δx=Δy=1.0, ε=0.03 (unless otherwise stated), β=1/9 and Re=100 for all of the simulations.

**Figure 1:**
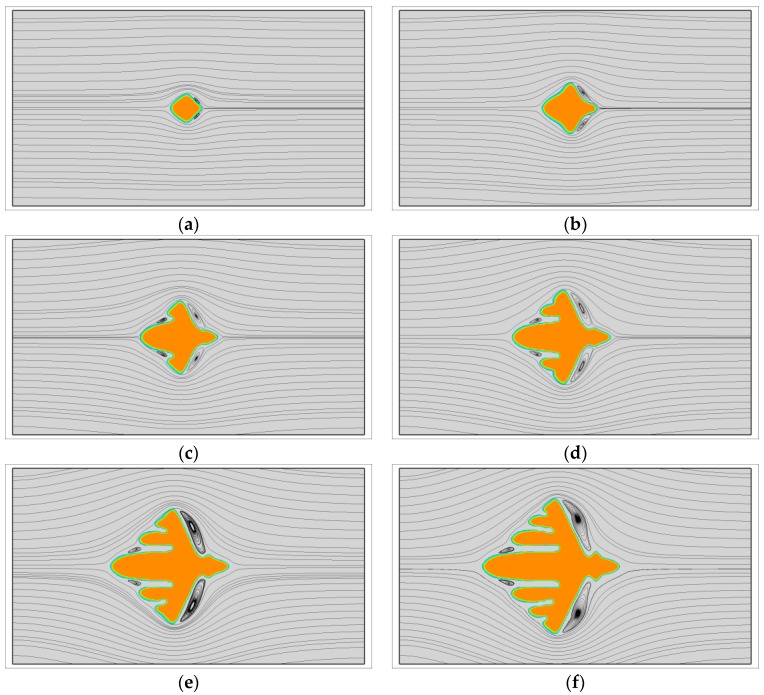
The growth of a dendrite under viscoelastic fluid flow with $u_{0}=1.0$ and We=1.0. In simulation, the dendritic morphology is triggered by the anisotropy with the number of mode j=4. (**a**) t=100; (**b**) t=200; (**c**) t=300; (**d**) t=400; (**e**) t=500; (**f**) t=600.

First, the growth of single crystals with viscoelastic fluid flow is investigated. At this situation, the fast melt flow prior to crystallization is not considered and all the crystals grow from point-like nuclei. Figure 1 shows the growth of a dendrite with $u_{0}=1.0$ and We=1.0. During the crystallization, the latent heat is transported from the upstream position to the downstream position by fluid flow. At this time, the crystal is surrounded by cool melt on the upstream side and warm melt on the downstream side. Therefore, the morphology of the crystal is quite different from the one obtained without flow. As we can see, the vertical branches tilt towards the upstream direction. On the upstream side, the growth of branches is rapid and all of the branches are growing nearly in the horizontal preferred direction. Conversely, on the downstream side the growth of sidebranches are completely prevented and the main branch grows at a relatively slow rate. This result is very similar with those obtained for metals [55]. Moreover, from the streamlines, the flow follows the interface at the early stage, but shortly strong vortexes are separated behind the vertical branches. Later on, small vortexes are even separated from the upstream main branch. It is not clear whether the contribution of those vortexes on the crystal morphology can be ignored, but at least they are unlikely the major contributors to the upstream preferred morphology.

**Figure 2:**
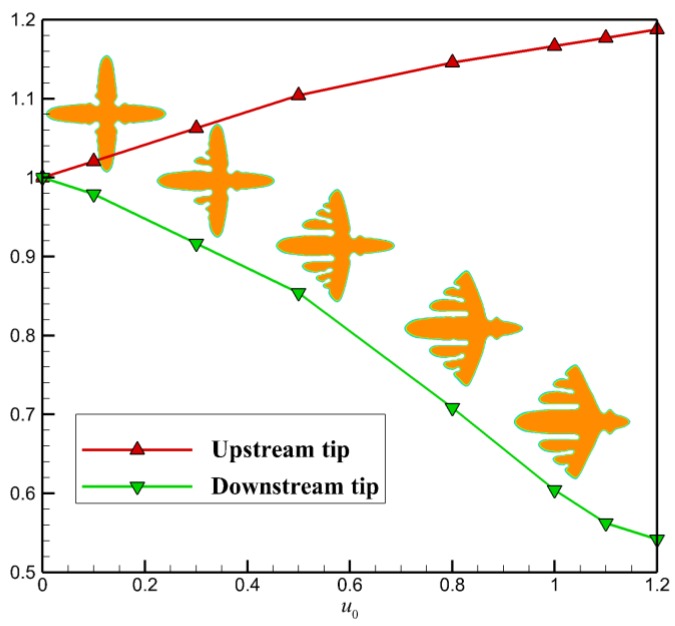
Quantitative study of the flow effect on the growth of the two main branches in the horizontal direction. The ratio between the averaged tip velocities with and without fluid flow is chosen as the measure. The two lines are the relative growth rates of the two branches at different inlet flow velocity.

Figure 2 shows a quantitative study of the flow effect on the growth of the two main branches in the horizontal direction. It is clear that faster flow results in a greater promoting rate on the growth of upstream tip and preventing rate on the growth of the downstream tip. At the flow condition $u_{0}=1.0$, the growth of upstream tip is promoted by nearly 16% and of the downstream tip is prevented by nearly 40%. Thus, by comparison, the crystal growth on the downstream side is more susceptible to fluid flow. From the crystal morphology arranged along the green line, we find that the side branches on the upstream side obtained with stronger flow are always more abundant.

**Figure 3:**
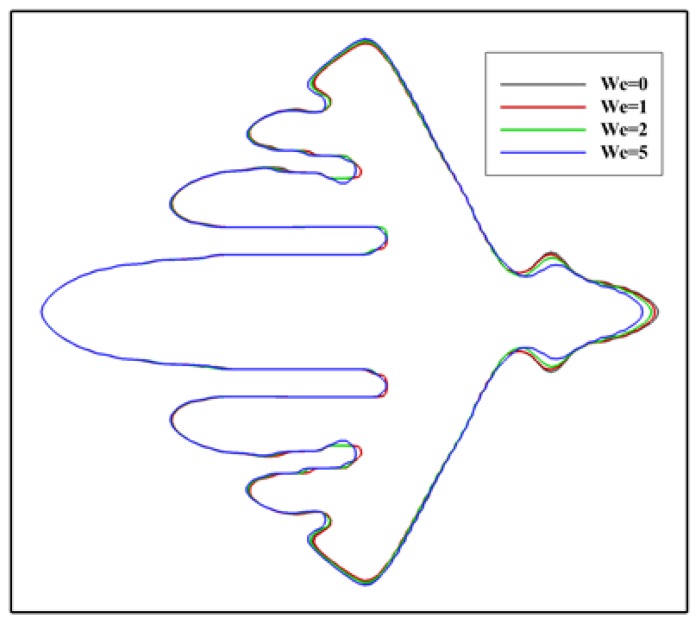
Crystal morphology at t=600 for different level of viscoelasticity. The Weissenberg number is changed from We=0 to We=5.0 and the inlet velocity is set as $u_{0}=1.0$. The different contours represent the solid/liquid interface position at different We.

In literature, the dendritic growth of metal crystals in shear flows has been studied for a long time. Newtonian fluid flows were considered in these studies. However, for polymers, the melt usually exhibits elasticity. Thus, it is reasonable to consider viscoelastic fluid flows in simulating the polymer crystallization under flow condition. At present, it is not clear to what extent the viscoelasticity affects the crystallization. Figure 3 shows the simulated crystal morphology at a different level of viscoelasticity. From the figure, the influence of viscoelasticity on the morphology can be distinguished, although it is not significant. It seems that the growth of the branch on the downstream side is more likely to be affected. Specifically, on the upstream side, it is unexpected that the growth of the main branch is not affected and only growth of the sidebranches is promoted. On the downstream side, the growth is prevented in a greater extent with stronger viscoelasticity. On the whole, these results indicate that the flow field and temperature distribution are changed little by viscoelastic stress during crystallization. For this reason, the Weissenberg number is fixed at We=1.0 for the following simulations. Note that the flow we considered is somewhat weak. Under stronger flows, the situation may be changed.

**Figure 4:**
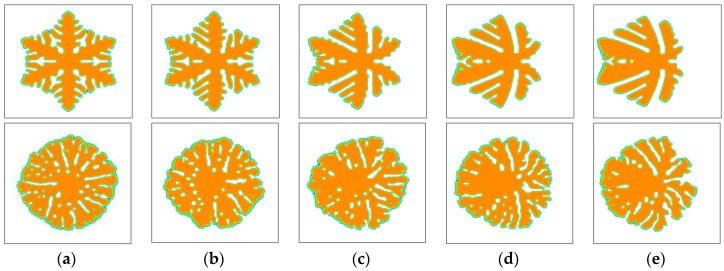
Simulated morphology of snowflake and seaweed crystals at t=600. The inlet velocity is changed from $u_{0}=0$ to $u_{0}=1.0$ and the viscoelastic parameter is set as We=1.0. The snowflake crystals and seaweed crystals are arranged in the top row and bottom row, respectively. In simulations, the snowflake morphology is triggered by the anisotropy with the number of mode j=6 and the seaweed morphology is triggered by a uniformly distributed noise added on the growing interface with the amplitude A=0.1 [50]. (**a**) $u_{0}=0$; (**b**) $u_{0}=0.2$; (**c**) $u_{0}=0.5$; (**d**) $u_{0}=0.8$; (**e**) $u_{0}=1.0$.

Except for the dendritic growth pattern, polymer crystals often exhibit many other growth patterns. Figure 4 shows the morphology variation of snowflake growth pattern and seaweed growth pattern under flow conditions. The simulated morphology is obviously affected by fluid flow. Similar to that observed for the dendritic growth pattern, the crystals possess nearly symmetric morphology in the absence of fluid flow and asymmetric morphology with fluid flow. Because of the flow effect, the crystal growth on the upstream side is still promoted and, on the downstream side, it is prevented. The vertical branches tilt towards the upstream direction so that the mass center of the whole crystal has a shift to the upstream side. Different growth patterns are affected by flow in a similar way but in different degrees. Generally, the growth pattern having more branching structure seems influenced less. For instance, the seaweed growth pattern that has a dense lamellar branching structure is the least influenced. The obtained crystal still has seaweed morphology. These results agree well with the experimental observations in literature [60]. Moreover, from the size of the crystals, one may find that the holistic growth rate of the crystals seems not affected much by flow. Therefore, it can be concluded that the flow effect on the kinetics of polymer crystallization mainly occurs in the nucleation stage. So far, plenty of experimental studies have ascertained the role of pre-shear in the kinetics of polymer crystallization [60,61,62]. It is clear that our conclusion from simulation is in accordance with the observations from experiments.

**Figure 5:**
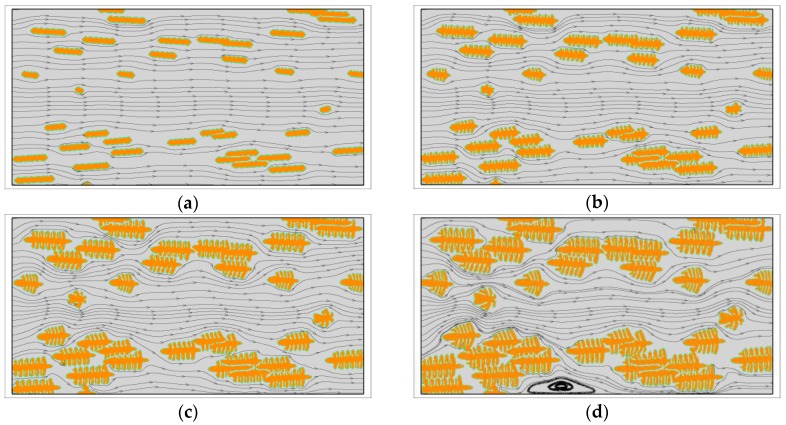
Simulated crystal growth under viscoelastic fluid flow. The simulation contains all the three steps. The strength of the fast flow prior to crystallization is set as $\hat{\gamma }=20$. The number and shape of the nuclei are calculated from the nucleation model given in Section 2.2. The viscoelastic fluid flow accompanying the crystal growth has parameters U=1.0 and We=1.0. The branching structure is triggered by the anisotropy with the number of mode j=6 for point-like nuclei and j=4 for oriented nuclei. (**a**) t=100; (**b**) t=200; (**c**) t=300; (**d**) t=400.

**Figure 6:**
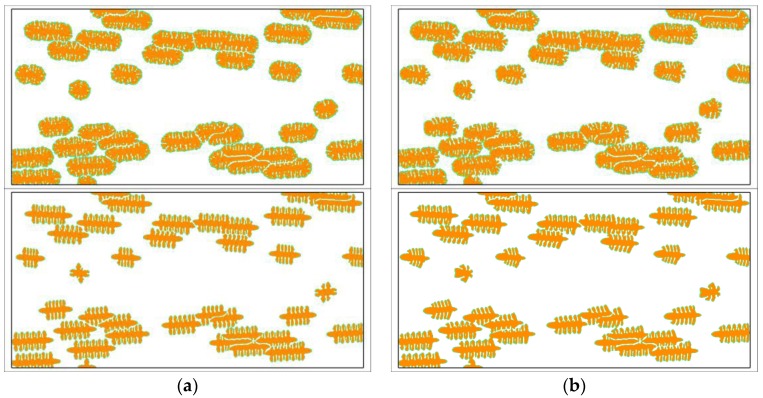
The comparisons between the simulated crystal morphology with and without fluid flow. The left and right columns are the results without and with flow, respectively. The results shown in the top row are triggered by noise and in the bottom row are triggered by the artificial anisotropy. (**a**) $u_{0}=0$; (**b**) $u_{0}=1.0$.

Researches about the growth of single crystals have important scientific values in predicting the growth mechanism of grains. However, in fact, there are almost no such single crystal morphologies in real materials. The knowledge getting from the study of single crystals only has guiding significance to the local crystallization behavior. In order to predict the flow effect on the holistic crystallization behavior of semi-crystalline polymers, the simulations should contain all of the three steps, which are: fast flow induced molecular structure, nucleation upon oriented molecular structure, and crystal growth with slow melt flow. Figure 5 shows the simulated crystal growth under viscoelastic fluid flow. It is clear that the crystals near the skin layer have greater aspect ratios and their orientations are nearly parallel to the flow direction. In addition, the nucleus density near the skin layer is greater than the core layer. Similar results have been obtained by experimental observations [51,61]. In our previous study [50], it has been found out that the fast flow prior to the crystallization is the primary cause of these phenomena. It is like we have observed from the results of single crystals the vertical branches tilting towards the upstream direction. In particular, because the melt in the skin layer flows slower than that in the core layer, the tilting degree of the crystals near the skin layer is not so obvious. The flow surrounding the crystals is in turn changed by crystallization. At the initial stage, the fluid flow is continuous so that all the streamlines are smooth. However, as the region occupied by crystals grows bigger, some streamlines begin to be interrupted by the sizeable crystals. The flow field gradually becomes very complex and unstable. In some places, the flow is stopped, and, in other places, vortexes are separated. Even so, the flow accompanying the crystal growth does not have a significant influence on the holistic crystallization behavior of semi-crystalline polymers. This can be clearly seen from the comparisons given in Figure 6.

Figure 6 shows some comparisons between the crystal morphology obtained with and without fluid flow. The results tell us that the holistic crystal morphology is not obviously changed by the viscoelastic fluid flow accompanying the crystal growth. Changes can only be observed in detail. By contrast, the simulation results for single crystals have exhibited great changes under the fluid flow with the same inlet velocity. That is to say, the flow effect on the morphology of single crystals is more prominent than on the morphology of bulk crystallizations. In real materials, bulk crystallizations are always the situations encountered. The simulations containing all the three steps are very close to the real crystallization procedure of semi-crystalline polymers [62]. From the simulation results, we conclude that the flow accompanying the crystal growth does not have a significant influence on the holistic crystal morphology, and the flow effect on the morphology also mainly occurs in the nucleation stage. Similar conclusions may also be drawn from the experimental observations [62]. As regards the influence of prior fluid flow on the crystal morphology, the readers are referred to our previous work [50] and some experimental observations [62].

Because of the numerical difficult of “high Weissenberg number problem”, the above simulations are limited to the crystal growth under a plane Poiseuille flow of FENE-P fluids. The possible improvements of our tool include extending the simulations to other type of flow, different polymer melt, 3D, etc. For these improvements, the main challenges are the numerical stability and computational cost. To ensure the stability, we can develop a more stable numerical method or use another polymer model. To settle the problem of high computational cost, we can propose a more efficient numerical method and use a faster computing platform. If these improvements are realized, more phenomena would be predicted by our model.

## 4. Conclusions

In this work, a multi-scale phase-field method has been presented for predicting the viscoelastic flow effect on the crystal growth of semi-crystalline polymers. To simplify the problem, the crystallization is divided into three parts. Firstly, in modeling the flow induced molecular structure, the FENE-P dumbbell model in microscale is coupled with the viscoelastic Navier–Stokes equations in macroscale to calculate the molecular orientation and stretch. Secondly, in modeling the nucleation upon oriented molecular structure, the influences of flow on nucleus shape and nucleus number are both taken into consideration. Concretely, the nucleus shape is related to the flow induced structures and nucleus number is linked to the flow induced stress. Thirdly, in modeling the crystal growth with melt flow, a massive mathematical model is established by coupling the phase-field with the temperature field, flow field and orientation field. The three parts together couple the mesoscopic crystallization with the microscopic orientation of chain segments and macroscopic viscoelastic melt flow. In solving the model, a semi-analytical method is adopted to avoid the numerical difficulty of “high Weissenberg number problem” in the first part, and an efficient fractional step method is used to reduce the computing complexity in the third part. Simulations are carried out for the crystallization of isotactic polystyrene under a plane Poiseuille flow. Results show that the flow effect on the morphology of single crystals is more prominent than on the morphology of bulk crystallizations. Specifically, the growth of single crystals including dendritic pattern, snowflake pattern and seaweed pattern all tilt toward the upstream direction. Meanwhile the growth of side branches gets promoted and inhibited on the upstream and downstream side, respectively. By comparison, the growth on the downstream side is more susceptible to fluid flow. For different growth patterns, the morphology having more branching structure seems less influenced by flow. Importantly, it is ascertained that the influence of viscoelasticity on the crystal morphology is not significant. As to the bulk crystallization in real materials, we find that flow does not have a significant influence on the holistic crystal morphology during the crystal growth stage and flow affects the morphology mainly through affecting the nucleation.

## Figures and Tables

**Figure A1:**
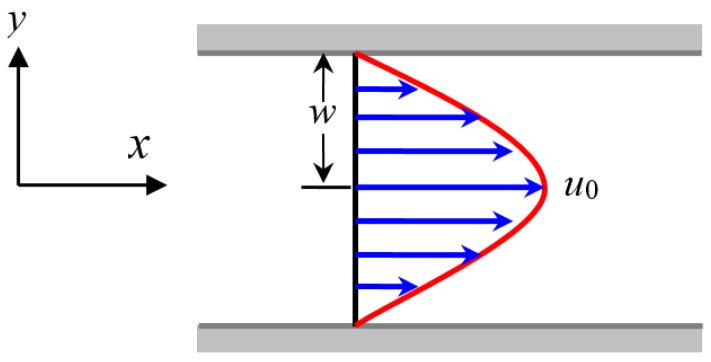
Schematic diagram of the steady-state plane Poiseuille flow.

## Appendix A. Nondimensionalization of the Models

In order to reduce the model parameters and facilitate the analysis, dimensionless forms of the PDE systems are presented. By introducing the parameters of the characteristic length for single crystals d, the mass diffusivity D and total viscosity of polymer $\eta_{0}=\eta_{s0}+\eta_{p0}$, and defining dimensionless variables

$$t^{*}=\frac{D}{d^{2}}t,x^{*}=\frac{x}{d},u^{*}=\frac{d}{D}u,p^{*}=\frac{d^{2}}{\eta_{0}D}p,\tau_{p}^{*}=\frac{d^{2}}{\eta_{0}D}\tau_{p}$$

and dimensionless parameters

$$\beta =\frac{\eta_{s0}}{\eta_{s0}+\eta_{p0}},Re=\frac{\rho D}{\eta_{0}},We=\lambda D/d^{2}$$

The dimensionless form of the PDE system (7)–(10) can be given by (the superscript is omitted):

(A1) ∇⋅u=0

(A2) $$Re(\frac{\partial u}{\partial t}+u\cdot \nabla u)=-\nabla p+\beta \Delta u+\nabla \cdot \tau_{p}$$

(A3) $$We\left(\frac{DC}{Dt}-{\left(\nabla u\right)}^{T}\cdot C-C\cdot (\nabla u)\right)=-\frac{C}{1-tr(C)/b}+I$$

(A4) $$\tau_{p}=\frac{1-\beta }{We}\left(\frac{C}{1-tr(C)/b}-I\right)$$

Here, the dimensionless parameter β represents the contribution rate of solvent viscosity to the total viscosity, the Reynolds number Re is the ratio of momentum forces to viscous forces quantifying the relative importance of these two types of forces for given flow conditions, and the Weissenberg number We compares the viscous forces to the elastic forces.

Moreover, if we further introduce the dimensionless temperature

$$T^{*}=\frac{T-T_{c}}{T_{m}-T_{c}}$$

with $T_{c}$ being the experimental temperature of crystallization, and dimensionless parameters

$${\tilde{\kappa }}_{0}=\frac{\kappa_{0}}{d},\alpha =\frac{k_{T}}{\rho C_{p}D},K=\frac{\Delta H}{(T_{m}-T_{c})C_{p}},Cp=\frac{C_{p}(T_{m}-T_{c})d^{2}}{D^{2}}$$

and estimate the mobility Γ from d and D as $\Gamma =D/d^{2}$. Then, the dimensionless form of the PDE system (26)–(31) is obtained as (the superscript is omitted):

(A5) $$\begin{array}{ll} \frac{\partial \psi }{\partial t}= & -(W\psi (\psi -\zeta )(\psi -\zeta_{0})-\kappa_{0}^{2}\nabla \cdot \left(\beta^{2}(\theta )\nabla \psi \right)\\ & +\kappa_{0}^{2}\frac{\partial }{\partial x}\left(\beta (\theta )\beta^{\prime }(\theta )\frac{\partial \psi }{\partial y}\right)-\kappa_{0}^{2}\frac{\partial }{\partial y}\left(\beta (\theta )\beta^{\prime }(\theta )\frac{\partial \psi }{\partial x}\right)) \end{array}$$

(A6) $$\frac{\partial T}{\partial t}+[1-\psi /\zeta_{0}]u\cdot \nabla T=\alpha \nabla^{2}T+K\frac{\partial \psi }{\partial t}+\frac{1}{ReCp}\tau :\nabla ([1-\psi /\zeta_{0}]u)$$

(A7) $$\nabla \cdot ([1-\psi /\zeta_{0}]u)=0$$

(A8) $$\begin{array}{cc} Re\left(\frac{\partial ([1-\psi /\zeta_{0}]u)}{\partial t}+[1-\psi /\zeta_{0}]u\cdot \nabla u\right) & =-[1-\psi /\zeta_{0}]\nabla p\\ +\beta (1+a_{0}\psi )\Delta ([1-\psi /\zeta_{0}]u) & +\nabla \cdot ([1-\psi /\zeta_{0}]\tau_{p}) \end{array}$$

(A9) $$We\left(\frac{DC}{Dt}-{\left(\nabla [1-\psi /\zeta_{0}]u\right)}^{T}\cdot C-C\cdot (\nabla [1-\psi /\zeta_{0}]u)\right)=-\frac{C}{1-tr(C)/b}+I$$

(A10) $$\tau_{p}=\frac{\left(1-\beta )(1+a_{0}\psi \right)}{We}\left(\frac{C}{1-tr(C)/b}-I\right)$$

where the dimensionless stress τ in Equation (A6) has the form

(A11) $$\tau =\beta (1+a_{0}\psi )\frac{\nabla u+\nabla u^{T}}{2}+\tau_{p}$$

## Appendix B. Method for the Molecular Structure Model

As is well known, numerical difficulty usually arises when solving the coupled system (A1)–(A4)—that is, the so called “high Weissenberg number problem” [63]. In order to avoid this kind of difficulty, only some simple flows with steady-state solutions are considered. Take the plane Poiseuille flow, for example. If we assume that the flow field always reaches its steady state during the induced period, then the final molecular configuration caused by such flow will be solved exactly. Consequently, the difficulty of “high Weissenberg number problem” will be avoided and the molecular configuration caused by strong flow can also be easily obtained.

For the steady-state plane Poiseuille flow illustrated in Figure A1, its velocity can be expressed as $u={\left(u,v\right)}^{T}={\left(u_{0}(1-y^{2}/w^{2}),0\right)}^{T}$, where $u_{0}$ is the velocity at the middle axis and w is the half width of the tube. Because the flow is in its steady state and the flow variables are invariant along the x-axis, the configuration tensor C is irrelevant to time t and space variable x. Therefore, we have

(A12) $$\begin{array}{l} \frac{DC}{Dt}-{\left(\nabla u\right)}^{T}\cdot C-C\cdot (\nabla u)\\ =\frac{\partial C}{\partial t}+(u\cdot \nabla )C-{\left(\nabla u\right)}^{T}\cdot C-C\cdot (\nabla u)=-\gamma \left(\begin{array}{cc} 2C_{12} & C_{22}\\ C_{22} & 0 \end{array}\right) \end{array}$$

where $\gamma =\partial u/\partial y=-2u_{0}y/w^{2}$. By inserting Equations (A12) into (A3), we get

(A13) $$\gamma We\left(\begin{array}{cc} 2C_{12} & C_{22}\\ C_{22} & 0 \end{array}\right)=\frac{C}{1-tr(C)/b}-I$$

that is,

(A14) $$2\gamma WeC_{12}=C_{11}/(1-tr(C)/b)-1$$

(A15) $$\gamma WeC_{22}=C_{12}/(1-tr(C)/b)$$

(A16) $$C_{22}/(1-tr(C)/b)-1=0$$

From Equations (A14)–(A16), it is easy to get

(A17) $$C=\frac{b}{b+2}I$$

when y=0 and

(A18) $$C_{22}=\frac{|\gamma |We\sqrt{6(b+2)}\left(K-\frac{1}{K}\right)}{6{\left(\gamma We\right)}^{2}}$$

(A19) $$C_{11}=b-(b+1)C_{22}$$

(A20) $$C_{12}=\gamma WeC_{22}^{2}$$

when y≠0, where

(A21) $$K=\sqrt[{3}]{\delta +\sqrt{\delta^{2}+1}}$$

(A22) $$\delta =\frac{54b|\gamma |We}{{\left(\sqrt{6(b+2)}\right)}^{3}}$$

If we insert $\gamma =-2u_{0}y/w^{2}$ into Equations (A18)–(A20), then the configuration tensor under the plane Poiseuille flow will be exactly solved. In order to facilitate the following discussion, the maximum shear rate of the plane Poiseuille flow is denoted by $\gamma_{m}=2u_{0}/w$, and a parameter $\hat{\gamma }=We\cdot \gamma_{m}$ is used to measure the relative flow strength.

## Appendix C. Method for the Crystal Growth Model

In 2D, the system (A5)–(A11) contains eight coupled PDEs. If those PDEs are solved by implicit schemes, great numerical difficulty will be encountered. To reduce the simulation difficulty, an explicit fractional step method is adopted. The component form of the semi-discretized system is as follows:

(A23) $$\begin{array}{ll} \frac{\psi^{n+1}-\psi^{n}}{\Delta t}= & -(W\psi (\psi -\zeta )(\psi -\zeta_{0})-\kappa_{0}^{2}\left(\frac{\partial }{\partial x}\left(\beta^{2}(\theta )\frac{\partial \psi }{\partial x}\right)+\frac{\partial }{\partial y}\left(\beta^{2}(\theta )\frac{\partial \psi }{\partial y}\right)\right)\\ & {+\kappa_{0}^{2}\frac{\partial }{\partial x}\left(\beta (\theta )\beta^{\prime }(\theta )\frac{\partial \psi }{\partial y}\right)-\kappa_{0}^{2}\frac{\partial }{\partial y}\left(\beta (\theta )\beta^{\prime }(\theta )\frac{\partial \psi }{\partial x}\right))}^{n} \end{array}$$

(A24) $$\begin{array}{ll} \frac{T^{n+1}-T^{n}}{\Delta t}+{\bar{u}}^{n}\frac{\partial T^{n}}{\partial x} & +{\bar{v}}^{n}\frac{\partial T^{n}}{\partial y}=\alpha {\left(\frac{\partial^{2}T}{\partial x^{2}}+\frac{\partial^{2}T}{\partial y^{2}}\right)}^{n}+K\frac{\psi^{n}-\psi^{n-1}}{\Delta t}\\ & +\frac{\beta (1+a_{0}\psi )}{ReCp}{\left({\left(\frac{\partial \bar{u}}{\partial x}\right)}^{2}+\frac{1}{2}{\left(\frac{\partial \bar{u}}{\partial y}+\frac{\partial \bar{v}}{\partial x}\right)}^{2}+{\left(\frac{\partial \bar{v}}{\partial y}\right)}^{2}\right)}^{n}\\ & +\frac{1}{ReCp}{\left(\frac{\partial \bar{u}}{\partial x}\tau_{11}+\left(\frac{\partial \bar{u}}{\partial y}+\frac{\partial \bar{v}}{\partial x}\right)\tau_{12}+\frac{\partial \bar{v}}{\partial y}\tau_{22}\right)}^{n} \end{array}$$

(A25) $$\begin{array}{ll} Re(\frac{\tilde{u}-{\bar{u}}^{n}}{\Delta t} & +{\bar{u}}^{n}\frac{\partial {\bar{u}}^{n}}{\partial x}+{\bar{v}}^{n}\frac{\partial {\bar{u}}^{n}}{\partial y})\\ & =\beta (1+a_{0}\psi ){\left(\frac{\partial^{2}\bar{u}}{\partial x^{2}}+\frac{\partial^{2}\bar{u}}{\partial y^{2}}\right)}^{n}+[1-\psi /\zeta_{0}]{\left(\frac{\partial \tau_{11}}{\partial x}+\frac{\partial \tau_{12}}{\partial y}\right)}^{n} \end{array}$$

(A26) $$\begin{array}{ll} Re(\frac{\tilde{v}-{\bar{v}}^{n}}{\Delta t} & +{\bar{u}}^{n}\frac{\partial {\bar{v}}^{n}}{\partial x}+{\bar{v}}^{n}\frac{\partial {\bar{v}}^{n}}{\partial y})\\ & =\beta (1+a_{0}\psi ){\left(\frac{\partial^{2}\bar{v}}{\partial x^{2}}+\frac{\partial^{2}\bar{v}}{\partial y^{2}}\right)}^{n}+[1-\psi /\zeta_{0}]{\left(\frac{\partial \tau_{12}}{\partial x}+\frac{\partial \tau_{22}}{\partial y}\right)}^{n} \end{array}$$

(A27) $${\left(\frac{\partial^{2}p}{\partial x^{2}}+\frac{\partial^{2}p}{\partial y^{2}}\right)}^{n+1}=\frac{Re}{\Delta t}\left(\frac{\partial \tilde{u}}{\partial x}+\frac{\partial \tilde{v}}{\partial y}\right)$$

(A28) $$\frac{u^{n+1}-\tilde{u}}{\Delta t}=-[1-\psi /\zeta_{0}]\frac{1}{Re}\frac{\partial p^{n+1}}{\partial x}$$

(A29) $$\frac{v^{n+1}-\tilde{v}}{\Delta t}=-[1-\psi /\zeta_{0}]\frac{1}{Re}\frac{\partial p^{n+1}}{\partial y}$$

(A30) $$\frac{C_{11}^{n+1}-C_{11}^{n}}{\Delta t}+{\left(\bar{u}\frac{\partial C_{11}}{\partial x}+\bar{v}\frac{\partial C_{11}}{\partial y}-2\frac{\partial \bar{u}}{\partial x}C_{11}-2C_{12}\frac{\partial \bar{u}}{\partial y}\right)}^{n}=\frac{1}{We}{\left(\frac{-C_{11}}{1-(C_{11}+C_{22})/b}+1\right)}^{n}$$

(A31) $$\frac{C_{12}^{n+1}-C_{12}^{n}}{\Delta t}+{\left(\bar{u}\frac{\partial C_{12}}{\partial x}+\bar{v}\frac{\partial C_{12}}{\partial y}-\frac{\partial \bar{u}}{\partial y}C_{22}-C_{11}\frac{\partial \bar{v}}{\partial x}\right)}^{n}=\frac{1}{We}{\left(\frac{-C_{12}}{1-(C_{11}+C_{22})/b}\right)}^{n}$$

(A32) $$\frac{C_{22}^{n+1}-C_{22}^{n}}{\Delta t}+{\left(\bar{u}\frac{\partial C_{22}}{\partial x}+\bar{v}\frac{\partial C_{22}}{\partial y}-2\frac{\partial \bar{v}}{\partial x}C_{12}-2C_{22}\frac{\partial \bar{v}}{\partial y}\right)}^{n}=\frac{1}{We}{\left(\frac{-C_{22}}{1-(C_{11}+C_{22})/b}+1\right)}^{n}$$

(A33) $$\tau_{11}^{n+1}=\frac{\left(1-\beta )(1+a_{0}\psi \right)}{We}{\left(\frac{C_{11}}{1-(C_{11}+C_{22})/b}-1\right)}^{n+1}$$

(A34) $$\tau_{12}^{n+1}=\frac{\left(1-\beta )(1+a_{0}\psi \right)}{We}{\left(\frac{C_{12}}{1-(C_{11}+C_{22})/b}\right)}^{n+1}$$

(A35) $$\tau_{22}^{n+1}=\frac{\left(1-\beta )(1+a_{0}\psi \right)}{We}{\left(\frac{C_{22}}{1-(C_{11}+C_{22})/b}-1\right)}^{n+1}$$

where Δt is the time step size, the superscript n means the corresponding variable taken its value at the time $t^{n}=n\Delta t$, and $\bar{u}$ represents the averaged velocity $[1-\psi /\zeta_{0}]u$. In simulations, all of the spatial derivatives are discretized by the standard central finite difference scheme. Note that the pressure Poisson Equation (A27) is the most time-consuming part in each time step. In this paper, a high efficient BiCGSTAB solver is used to reduce the cost.

For the phase-field Equation (A23), the model parameters W and $\kappa_{0}$ can be determined through experimentally measurable quantities. They may be calculated by [22]

(A36) $$W=6\frac{\Delta H}{RT\zeta_{0}^{3}}\left(\frac{T_{m}-T}{T_{m}^{0}}\right){\left(\frac{\zeta_{0}}{2}-\zeta \right)}^{-1}$$

(A37) $$\kappa_{0}=6\frac{\sigma }{nRT}{\left(\frac{2}{W}\right)}^{1/2}$$

with ΔH being the latent heat, R the gas constant, σ the surface free energy per unit area, and n the amount of substance of polymer monomers per unit volume. Finally, the unstable energy barrier ζ, which increases with temperature, may be determined by [25]

(A38) $$\zeta =\left(1+\frac{2}{\pi }\arctan(kT)\right)\frac{4\zeta_{0}\hat{\psi }-3{\hat{\psi }}^{2}}{6\zeta_{0}-4\hat{\psi }}$$

where $\hat{\psi }$ is estimated as $\hat{\psi }=\zeta_{0}(T_{m}^{0}-T_{m})/(T_{m}^{0}-T)$ and k is a dimensionless coefficient inversely proportional to the supercooling. In this work, it is simply evaluated as k=K.

## Appendix D. Summarization of the Whole Simulation Process

For the steady-state plane Poiseuille flow, the whole simulation process can be summarized as follows:

**Step 1: Fast Flow Induced Molecular Structure**

For given $\hat{\gamma }$, compute the configuration tensor C by Equations (A17)–(A20).

**Step 2: Nucleation upon Oriented Molecular Structure**

(1) For given $T_{c}$, calculate the density of activated nuclei N by Equations (11)–(14);
(2) Determine the center of each nucleus in the simulation area by a random number generator;
(3) Calculate the orientation and aspect ratio of each nucleus by Equations (11)–(20) according to the molecular configuration at the position where the nucleus is located;
(4) Generate the oriented nuclei upon the simulation lattice;

**Step 3: Crystal Growth with Slow Melt Flow**

(1) Given model parameters and initial conditions for the crystal growth model;
(2) For given $\psi^{n}$ and $T^{n}$, compute $\psi^{n+1}$ by Equation (A23);
(3) For given $T^{n}$, $\psi^{n}$, $\psi^{n-1}$, ${\bar{u}}^{n}=[1-\psi^{n}/\zeta_{0}]u^{n}$ and $\tau_{p}^{n}$, compute $T^{n+1}$ by Equation (A24);
(4) For given ${\bar{u}}^{n}\;=\;[1-\psi^{n}/\zeta_{0}]u^{n}$, $\psi^{n}$ and $\tau_{p}^{n}$, compute the intermediate velocity $\tilde{u}$ by Equations (A25) and (A26);
(5) For given $\tilde{u}$, compute the pressure $p^{n+1}$ by Equation (A27);
(6) For given $p^{n+1}$, correct the velocity $u^{n+1}$ by Equations (A28) and (A29);
(7) For given $C^{n}$ and ${\bar{u}}^{n}\;=\;[1-\psi^{n}/\zeta_{0}]u^{n}$, compute $C^{n+1}$ by Equations (A30)–(A32);
(8) Compute $\tau_{p}^{n+1}$ from $C^{n+1}$ by Equations (A33)–(A35);
(9) If the terminal time is not reached, update n by n+1 and return to (2).

Note that the physical problem we considered is first putting the hot polymer melt into a fast simple flow for a period of time, and then decreasing the flow to a large extent and cooling the melt to a scheduled crystallization temperature quite rapidly. Thus, the flow parameters used in the first step are generally different from those used in the third step. Moreover, to keep it simple in this paper, we assume that the ordered structures induced by the fast flow would be completely retained after the weakening of flow until the nucleation takes place. Based on this assumption, the transient process from the fast melt flow to the slow melt flow may be ignored.

## References

[1] Janeschitz-Kriegl H., Ratajski E. (2010). Some fundamental aspects of the kinetics of flow-induced crystallization of polymers. Colloid Polym. Sci..

[2] Ratajski E., Janeschitz-Kriegl H. (2012). Flow-induced crystallization in polymer melts: On the correlation between nucleation and specific work. Polym. Bull..

[3] Liu S.Y., Zhang F.F., Zheng G.Q., Dai K., Liu C.T., Shen C.Y., Guo J.Z. (2016). Direct microscopic observation of shish-kebab structuren high-temperature electrospun iPP fibers. Mater. Lett..

[4] Kumaraswamy G., Issaian A.M., Kornfield J.A. (1999). Shear-Enhanced Crystallization in Isotactic Polypropylene. 1. Correspondence between in Situ Rheo-Optics and ex Situ Structure Determination. Macromolecules.

[5] Zhou Y.G., Turng L.S., Shen C.Y. (2010). Morphological evolution and orientation development of stretched iPP films: Influence of draw ratio. J. Polym. Sci. B Polym. Phys..

[6] Hsiao B.S., Yang L., Somani R.H., Avila-Orta C.A., Zhu L. (2005). Unexpected shish-kebab structure in a sheared polyethylene melt. Phys. Rev. Lett..

[7] Rong Y., He H.P., Cao W., Shen C.Y., Chen J.B. (2013). Multi-scale molding and numerical simulation of the flow-induced crystallization of polymer. Comput. Mater. Sci..

[8] Mu Y., Zhao G.Q., Chen A.B., Dong G.W., Li S. (2014). Numerical investigation of the crystallization and orientation behavior in polymer processing with a two-phase model. Comput. Chem. Eng..

[9] Spina R., Spekowius M., Hopmann C. (2016). Multiphysics simulation of thermoplastic polymer crystallization. Mater. Des..

[10] Anwar M., Schilling T. (2015). Crystallization of polyethylene: A molecular dynamics simulation study of the nucleation and growth mechanisms. Polymer.

[11] Yamamoto T. (2013). Molecular dynamics of polymer crystallization revisited: Crystallization from the melt and the glass in longer polyethylene. J. Chem. Phys..

[12] Baig C., Edwards B.J. (2010). Atomistic simulation of crystallization of a polyethylene melt in steady uniaxial extension. J. Non-Newton. Fluid Mech..

[13] Nie Y.J., Zhang R.J., Zheng K.S., Zhou Z.P. (2015). Nucleation details of nanohybrid shish-kebabs in polymer solutions studied by molecular simulations. Polymer.

[14] Hu W., Frenkel D., Mathot V.B.F. (2002). Simulation of shish-kebab crystallite induced by a single prealigned macromolecule. Macromolecules.

[15] Zhou Y.G., Turng L.S., Shen C.Y. (2010). Modeling and prediction of morphology and crystallinity for cylindrical-shaped crystals during polymer processing. Polym. Eng. Sci..

[16] Ruan C.L., Liu C.T., Zheng G.Q. (2015). Monte Carlo simulation for the morphology and kinetics of spherulites and shish-kebabs in isothermal polymer crystallization. Math. Probl. Eng..

[17] Graham R.S., Olmsted P.D. (2009). Coarse-grained simulations of flow-induced nucleation in semicrystalline polymers. Phys. Rev. Lett..

[18] Jolley K., Graham R.S. (2011). A fast algorithm for simulating flow-induced nucleation in polymers. J. Chem. Phys..

[19] Graham R.S. (2014). Modelling flow-induced crystallisation in polymers. Chem. Commun..

[20] Piorkowska E., Billon N., Haudin J.M., Gadzinowska K. (2005). Spherulitic Structure development during crystallization in confined space II. Effect of spherulite nucleation at borders. J. Appl. Polym. Sci..

[21] Raabe D., Godara A. (2005). Mesoscale simulation of the kinetics and topology of spherulite growth during crystallization of isotactic polypropylene (iPP) by using a cellular automaton. Model. Simul. Mater. Sci. Eng..

[22] Xu H.J., Matkar R., Kyu T. (2005). Phase-field modeling on morphological landscape of isotactic polystyrene single crystals. Phys. Rev. E.

[23] Wang D., Shi T.F., Chen J.Z. (2008). Simulated morphological landscape of polymer single crystals by phase field model. J. Chem. Phys..

[24] Wang X.D., Ouyang J., Su J., Zhou W. (2014). Phase field modeling of the ring-banded spherulites of crystalline polymers: The role of thermal diffusion. Chin. Phys. B.

[25] Wang X.D., Ouyang J., Su J., Zhou W. (2013). A phase-field model for simulating various spherulite morphologies of semi-crystalline polymers. Chin. Phys. B.

[26] Huang T., Kamal M.R. (2000). Morphological Modeling of Polymer Solidification. Polym. Eng. Sci..

[27] Criscione A., Kintea D., Tuković Ž., Jakirlić S., Roisman I.V., Tropea C. (2013). Crystallization of supercooled water: A level-set-based modeling of the dendrite tip velocity. Int. J. Heat Mass Transf..

[28] López J., Gómez P., Hernández J. (2010). A volume of fluid approach for crystal growth simulation. J. Comput. Phys..

[29] Liu Z.J., Ouyang J., Zhou W., Wang X.D. (2015). Numerical simulation of the polymer crystallization during cooling stage by using level set method. Comput. Mater. Sci..

[30] Kobayashi R. (1993). Modeling and numerical simulations of dendritic crystal growth. Physica D.

[31] Karma A., Rappel W.J. (1998). Quantitative phase-field modeling of dendritic growth in two and three dimensions. Phys. Rev. E.

[32] Boettinger W.J., Warren J.A., Beckermann C., Karma A. (2002). Phase-field simulation of solidification. Annu. Rev. Mater. Res..

[33] Gránásy L., Pusztai T., Warren J.A. (2004). Modelling polycrystalline solidification using phase field theory. J. Phys. Condens. Mat..

[34] Gránásy L., Rátkai L., Szállás A., Korbuly B., Tóth G., Környei L., Pusztai T. (2014). Phase-field modeling of polycrystalline solidification: From needle crystals to spherulites-A Review. Metall. Mater. Trans. A.

[35] Wang X.D., Zhang H.X., Zhou W., Ouyang J. (2017). A 3D phase-field model for simulating the crystal growth of semi-crystalline polymers. Int. J. Heat Mass Transf..

[36] Xu H.J., Keawwattana W., Kyu T. (2005). Effect of thermal transport on spatiotemporal emergence of lamellar branching morphology during polymer spherulitic growth. J. Chem. Phys..

[37] Wang D., Jin Z.K., Xing Y., Gao H., Wang X.K. (2013). Simulated rhythmic growth of targeted single crystal by polymer phase-field model. Comput. Mater. Sci..

[38] Wang X.D., Ouyang J., Su J., Zhou W. (2014). Investigating the role of oriented nucleus in polymer shish-kebab crystal growth via phase-field method. J. Chem. Phys..

[39] Tong X., Beckermann C., Karma A., Li Q. (2001). Phase-field simulations of dendritic crystal growth in a forced flow. Phys. Rev. E.

[40] Sun D.K., Zhu M.F., Pan S.Y., Yang C.R., Raabe D. (2011). Lattice Boltzmann modeling of dendritic growth in forced and natural convection. Comput. Math. Appl..

[41] De Gennes P.G. (1974). Coil-stretch transition of dilute flexible polymers under ultrahigh velocity gradients. J. Chem. Phys..

[42] Azzurri F., Alfonso G.C. (2008). Insights into formation and relaxation of shearinduced nucleation precursors in isotactic polystyrene. Macromolecules.

[43] Cavallo D., Azzurri F., Balzano L., Funari S.S., Alfonso G.C. (2010). Flow memory and stability of shear-induced nucleation precursors in isotactic polypropylene. Macromolecules.

[44] Hamad F.G., Colby R.H., Milner S.T. (2015). Lifetime of flow-induced precursors in isotactic polypropylene. Macromolecules.

[45] Guo X., Isayev A.I., Guo L. (1999). Crystallinity and microstructure in injection molding of isotactic polypropylenes Part 1: A new approach to modeling and model parameters. Polym. Eng. Sci..

[46] Boutaousa M., Bourgin P., Zinet M. (2010). Thermally and flow induced crystallization of polymers at low shear rate. J. Non-Newton. Fluid Mech..

[47] Yu F.Y., Zhang H.B., Wang Z.G., Yu W., Zhou C.X. (2010). Overshoots in stress and free energy change during the flow-induced crystallization of polymeric melt in shear flow. Chin. J. Polym. Sci..

[48] Upadhyay R.K., Isayev A.I., Shen S.F. (1981). Transient shear flow behavior of polymeric fluids according to the Leonov model. Rheol. Acta.

[49] Herrchen M., Öttinger H.C. (1997). A detailed comparison of various FENE dumbbell models. J. Non-Newton. Fluid Mech..

[50] Wang X.D., Ouyang J., Zhou W., Liu Z.J. (2016). A phase field technique for modeling and predicting flow induced crystallization morphology of semi-crystalline polymers. Polymers.

[51] Koscher E., Fulchiron R. (2002). Influence of shear on polypropylene crystallization: Morphology development and kinetics. Polymer.

[52] Charbon C., Swaminarayan S. (1998). A multiscale model for polymer crystallization. II. Solidification of a macroscopic part. Polym. Eng. Sci..

[53] Pantani R., De Santis F., Speranza V., Titomanlio G. (2014). Modelling morphology evolution during solidification of IPP in processing conditions. AIP Conf. Proc..

[54] Anderson D.M., McFadden G.B., Wheeler A.A. (2000). A phase-field model of solidification with convection. Phys. D.

[55] Tönhardt R., Amberg G. (1998). Phase-field simulation of dendritic growth in a shear flow. J. Cryst. Growth.

[56] Tönhardt R., Amberg G. (2000). Dendritic growth of randomly oriented nuclei in a shear flow. J. Cryst. Growth.

[57] Beckermann C., Diepers H.J., Steinbach I., Karma A., Tong X. (1999). Modeling melt convection in phase-field simulations of solidification. J. Comput. Phys..

[58] Pantani R., Coccorullo I., Speranza V., Titomanlio G. (2005). Modeling of morphology evolution in the injection molding process of thermoplastic polymers. Prog. Polym. Sci..

[59] Katayama K., Yoon M.G. (1985). Polymer Crystallization in Melt Spinning: Mathematical Simulation.

[60] Najafi N., Heuzey M.C., Carreau P., Therriault D. (2015). Quiescent and shear-induced crystallization of linear and branched polylactides. Rheol. Acta.

[61] Zhang C.G., Hu H.Q., Wang D.J., Yan S.K., Han C.C. (2005). In situ optical microscope study of the shear-induced crystallization of isotactic polypropylene. Polymer.

[62] Sun T., Chen F., Dong X., Zhou Y., Wang D., Han C.C. (2009). Shear-induced orientation in the crystallization of an isotactic polypropylene nanocomposite. Polymer.

[63] Su J., Ouyang J., Wang X.D., Yang B.X. (2013). Lattice Boltzmann method coupled with the Oldroyd-B constitutive model for a viscoelastic fluid. Phys. Rev. E.

